# Artificial intelligence in pediatric arrhythmias: current landscape, unique challenges, and translational perspectives

**DOI:** 10.3389/fped.2026.1932796

**Published:** 2026-07-31

**Authors:** Hailin Jia, Wenjing Zhu, Jianli Lv

**Affiliations:** 1 Department of Pediatric Cardiology, Shandong Provincial Hospital Affiliated to Shandong First Medical University, Jinan, China; 2 Department of Pulmonary and Critical Care Medicine, Shandong Provincial Hospital Affiliated to Shandong First Medical University, Jinan, China

**Keywords:** arrhythmias, artificial intelligence, deep learning, electrocardiography, machine learning, pediatric cardiology, precision medicine, wearable devices

## Abstract

**Background:**

The interpretation of pediatric electrocardiograms (ECGs) and management of childhood arrhythmias represent specialized clinical disciplines complicated by age-dependent physiological evolution. While artificial intelligence (AI) has transformed adult cardiology, its application to pediatric electrophysiology remains largely in the research phase.

**Objective:**

This review critically appraises the current evidence, methodological rigor, clinical readiness, and translational challenges of AI in pediatric arrhythmia detection, risk stratification, and management.

**Methods:**

A synthesis of contemporary literature was conducted, evaluating machine learning (ML), deep learning (DL), large language models (LLMs), wearable sensors, and intensive care monitoring across pediatric cohorts.

**Main findings:**

While purpose-built DL models demonstrate strong diagnostic performance for specific electrical phenotypes—such as Wolff-Parkinson-White (WPW) syndrome, long QT syndrome (LQTS), and neonatal bradycardia—the vast majority of published tools remain unvalidated retrospective proofs-of-concept. Current AI algorithms analyze isolated ECG waveforms under curated conditions and cannot replace holistic clinical evaluations incorporating patient history, family screening, genetics, and multi-modality diagnostic testing. Significant barriers persist, including pervasive data scarcity, lack of prospective external validation, limited saliency map reproducibility in Explainable AI (XAI), and uncalibrated false alarms. Furthermore, adult-trained algorithms and general-purpose LLMs yield unacceptable diagnostic error rates when applied to children.

**Conclusions:**

AI holds promise for enhancing pediatric arrhythmia care, but clinical integration requires moving beyond isolated performance metrics. Future progress hinges on prospective multicenter validation, privacy-preserving federated learning, multimodal data integration, and explicit definition of AI's role as a clinical decision-support tool within real-world workflows.

## Introduction

Pediatric arrhythmias encompass a broad spectrum of electrical conduction disorders, ranging from benign premature contractions to life-threatening channelopathies and post-operative intra-atrial re-entry tachycardias (IART) (1). Prompt recognition is crucial to prevent sudden cardiac death (SCD) in the young. However, pediatric electrocardiography is inherently complex: resting heart rate, conduction velocity, and electrical axes undergo non-linear changes from neonates through adolescents (2). Consequently, distinguishing physiological variation from subtle pathology requires specialized electrophysiological expertise that is often scarce in primary care, emergency, or resource-limited settings.

Over the past decade, artificial intelligence (AI)—including deep neural networks (DNNs)—has generated paradigm shifts in adult cardiovascular medicine, demonstrating robust proficiency in arrhythmia detection, structural assessment, and event prediction (3–6). Conversely, pediatric electrophysiology has lagged behind. Direct transfer of adult-derived models (e.g., trained on the adult MIT-BIH database) to pediatric populations leads to systematic misclassification due to age-dependent physiological mismatches (7).

Crucially, while early literature highlighted the high diagnostic accuracy of experimental pediatric AI algorithms, enthusiasm must be tempered by rigorous critical appraisal. Most available pediatric AI tools remain retrospective research constructs tested on pre-filtered datasets. In real-world practice, expert clinicians interpret cardiac electrical activity not in isolation, but by synthesizing clinical history, physical examination, family screening, laboratory data, imaging, and serial ECGs. This narrative review critically evaluates the current evidence base for AI in pediatric arrhythmias, delineates proof-of-concept models from clinically ready tools, addresses key methodological limitations, and outlines realistic pathways for integrating AI into established pediatric clinical workflows.

## Overview of artificial intelligence techniques and methodological quality

### AI paradigms in pediatric electrophysiology

The technical landscape in pediatric arrhythmia analysis spans conventional machine learning (ML) and deep learning (DL) paradigms. Conventional ML algorithms—such as Support Vector Machines (SVM), Random Forests (RF), and Logistic Regression (LR)—rely on manually engineered feature extraction (e.g., QTc intervals, R-R variability, *P*-wave duration). These explicit inputs render conventional ML inherently interpretable and robust on smaller datasets (7, 8). However, manually feature-engineered models are susceptible to measurement errors in noisy pediatric ECGs.

In contrast, DL architectures—including Convolutional Neural Networks (CNNs) for spatiotemporal waveform features and Recurrent Neural Networks (RNNs/LSTMs) for long-term temporal trends—autonomously extract high-dimensional patterns directly from raw signal data (9, 10). While DL models frequently achieve superior diagnostic performance metrics on curated datasets, their internal decision-making processes remain opaque (“black box”), precluding straightforward physiological verification. General-purpose Large Language Models (LLMs) and multimodal vision-language models (e.g., GPT-4o, Gemini) have also been explored; however, recent evaluations confirm that these models exhibit poor diagnostic specificity around thirty percent and near-unity positive likelihood ratios on pediatric ECGs, demonstrating a systematic tendency toward false-positive overcalling that precludes standalone clinical deployment (11–13).

### Critical appraisal of methodological quality

A rigorous evaluation of published pediatric AI literature reveals consistent methodological vulnerabilities that must be recognized before clinical translation (Table 1). Over ninety percent of published pediatric AI models are retrospective, single-center studies trained on enriched cohorts with higher disease prevalence than general clinical populations, a spectrum bias that systematically inflates sensitivity and positive predictive value. Rare pediatric channelopathies like Brugada syndrome and Catecholaminergic Polymorphic Ventricular Tachycardia suffer from severe data scarcity, forcing models trained on small sample sizes to risk memorizing noise rather than learning generalizable electrophysiological patterns. Furthermore, fewer than fifteen percent of published models undergo validation on independent multicenter datasets from diverse geographic or ethnic populations, and virtually none have been subjected to prospective clinical trials or regulatory evaluation for FDA clearance or CE mark approval. Finally, algorithm benchmarks are frequently derived from clear, artifact-free ECG recordings, ignoring real-world pediatric challenges such as motion artifacts, lead displacement, uncooperative infant behavior, and wandering baselines.

**Table 1:** Taxonomy and evidence maturity matrix of AI applications in pediatric arrhythmias.

Application Domain	Key AI Paradigm/Models	Methodological Strengths	Methodological Limitations & Barriers	Level of Evidence & Clinical Readiness
LQTS & Channelopathies	pedQTNet, AI-ECG (CNNs), DNN mutation detectors	Accurate QTc estimation (MAE ∼18.8 ms); sub-visual detection of concealed LQTS (AUC 0.86)	Retrospective; cannot evaluate dynamic variability; does not replace challenge testing or genetics	Proof-of-Concept/Experimental (Unvalidated for standalone triage)
WPW & Pre-excitation	Multimodal CNNs (12-lead ECG + Chest x-ray)	High localization accuracy (∼84%); vast training cohorts	Evaluated on curated ECGs; lacks prospective validation for ablation outcome prediction	Near-Clinical Validation (Potential decision-support; requires trial)
Brugada Syndrome	Two-stage Transfer Learning (CNN-BiLSTM)	Overcomes data scarcity via RBBB pre-training (AUC 0.96)	Ignores temporal/fever-induced variability of Type 1 pattern; single resting ECG limitation	Early Proof-of-Concept (Requires longitudinal validation)
Neonatal Intensive Care	Autoencoders, 1D-CNN, LSTM	Sensitivity 0.992; reduces false alarms by ∼36%	Output correction tested offline; lacking evidence for improved clinical outcomes or reduced mortality	Translational Research (Needs real-time bedside clinical trial)
CHD & Postoperative Arrhythmias	Random Forest, XGBoost, DL-CMR segmentation	Predicts pmVSD closure heart block & rToF VT risk	Single-center datasets; specific to operative device types/surgical techniques	Exploratory/Research Phase
Wearable Surveillance	DNNs in smartwatch/textile platforms	Continuous monitoring; detects SVT/PVCs in active children	High motion artifact rate; low pediatric compliance; unapproved for pediatric diagnostic use	Commercially Available/Clinically Unvalidated (Consumer-grade)

To understand the translation readiness of AI tools, published applications must be stratified by their methodological evidence maturity. Experimental proof-of-concept models include deep learning mutation detectors, AI-ECG concealed channelopathy identifiers, and postmortem digital histopathology pipelines, all of which remain restricted to research contexts due to single-center retrospective designs. Translational research tools—such as NICU false-alarm reduction algorithms and pediatric wearable telemetry platforms—demonstrate feasibility but lack prospective trials proving clinical outcome improvements or formal regulatory approvals for pediatric diagnostic use. Near-clinical validation constructs, exemplified by large-scale deep learning models for pre-excitation detection trained on extensive institutional cohorts, represent the closest step toward decision-support deployment, yet still mandate prospective human-in-the-loop clinical trials prior to routine workflow integration.

## Current applications in specific pediatric arrhythmias

### Long QT syndrome and inherited channelopathies

Congenital LQTS requires precise corrected QT (QTc) measurement, a task rendered difficult in children by elevated resting heart rates and age-dependent T-wave morphology. Deep learning models, such as pedQTNet, have demonstrated low mean absolute error (18.8 ms) in QTc estimation across pediatric cohorts (10). Beyond interval estimation, AI-enhanced ECGs (AI-ECG) have identified sub-visual morphological signatures in concealed LQTS (genetically confirmed LQT1–3 with normal resting QTc), achieving an AUC of 0.863 (14, 15). Furthermore, neural network analysis of 24-hour Holter recordings has identified maximal QTc as a key diagnostic metric and QTc dispersion (QTcd) as a prognostic marker for adverse arrhythmic events (16).

Despite these promising metrics, significant clinical boundaries must be emphasized. AI models operate strictly on static electrical phenotypes captured during brief recording windows and cannot replace comprehensive clinical evaluation, which remains the cornerstone of channelopathy management. Crucially, AI-ECG cannot substitute for genetic testing to identify specific pathogenic variants and guide gene-specific pharmacotherapy. Nor can it replace family cascade screening to uncover silent familial inheritance, provocative treadmill exercise testing or epinephrine challenge protocols to unmask latent QT prolongation, or multidimensional clinical risk stratification incorporating syncope history and sex-specific post-pubertal risks.

### Brugada syndrome and intermittent electrophysiological patterns

In rare channelopathies like Brugada syndrome, data scarcity presents a major obstacle. Liu et al. addressed this via a two-stage transfer learning architecture, pre-training a CNN-BiLSTM on right bundle branch block (RBBB) ECGs prior to fine-tuning on Brugada cases, achieving an AUC of 0.96 on retrospective testing (17).

While architecturally novel, single ECG-based AI models possess fundamental physiological limitations in Brugada syndrome. The diagnostic Type 1 Brugada pattern is notorious for extreme temporal variability, often remaining entirely latent until unmasked by fever, vagal tone, sodium channel blocker provocation, or specific electrolyte shifts. An AI model analyzing a single baseline resting ECG recorded during a silent phase will inevitably return a false-negative result. Consequently, AI assessment cannot replace high-unipolar lead placement (2nd/3rd intercostal spaces), longitudinal monitoring, fever-state ECG acquisition, or pharmacological challenge testing.

### Wolff-Parkinson-White syndrome and pre-excitation

In WPW syndrome, pre-procedural accessory pathway (AP) localization is critical for catheter ablation planning. Recent DL models integrating 12-lead ECGs with chest radiographs achieved an 84% localization accuracy (AUC 0.92) by accounting for pediatric cardiac rotation and anatomical variability (18, 19). In a landmark multicenter study, Mayourian et al. trained a CNN on over 580,000 pediatric ECGs, demonstrating high diagnostic accuracy for pre-excitation (AUC 0.99) that outperformed standard commercial rule-based software (9).

While published studies focus heavily on AP localization, the clinical utility of AI in Wolff-Parkinson-White syndrome must expand into actionable procedural and prognostic domains. Future models should focus on non-invasive risk stratification for SCD by identifying high-risk AP conduction properties, such as short anterograde effective refractory periods during rapid pre-excited atrial fibrillation. Furthermore, AI should be leveraged for procedural planning to predict mechanical pathway conduction block, assess proximity to the normal conduction system, estimate the risk of transient or permanent heart block, and calculate post-ablation recurrence rates based on subtle baseline delta-wave morphology and electroanatomical mapping data.

### Neonatal monitoring and intensive care

In the Neonatal Intensive Care Unit (NICU), premature infants experience frequent apnea of prematurity and secondary bradycardia. Bedside monitors generate excessive false alarms (up to 80%), inducing severe caregiver alarm fatigue. Autoencoders and 1D-CNN architectures equipped with signal quality indices have reduced false bradycardia alarms by ∼36% while maintaining high sensitivity (0.992) (20–22). Additionally, machine learning models analyzing heart rate variability (HRV) have provided non-invasive estimates of autonomic maturation in preterm infants (23).

A critical gap in current neonatal AI literature is the implicit assumption that reducing false alarms directly equates to improved patient outcomes. To date, there is a total absence of prospective clinical evidence demonstrating that AI-driven alarm suppression reduces neonatal morbidity, hypoxic-ischemic brain injury, length of NICU stay, or mortality. Future research must move beyond offline signal processing validation to prospective clinical trials measuring tangible patient-centered endpoints and healthcare worker cognitive workload.

## Postoperative, congenital heart disease, and complex atrial arrhythmias

### Postoperative risk prediction in structural CHD

Patients with congenital heart disease (CHD) harbor complex, lifelong arrhythmic substrates. Machine learning classifiers (Random Forest, XGBoost) integrating surgical parameters, fluoroscopy duration, defect-to-device ratios, and patient weight have successfully predicted early postoperative complete heart block and tachyarrhythmias following transcatheter perimembranous ventricular septal defect (pmVSD) closure (24). Similarly, deep learning analysis of cardiac magnetic resonance (CMR) imaging in repaired Tetralogy of Fallot (rToF) has automated right ventricular strain and right atrial volumetric quantification, predicting long-term sustained ventricular tachycardia (VT) and cardiovascular mortality (25).

### Complex atrial arrhythmias in CHD (Fontan, IART, and atrial flutter)

The arrhythmic burden in pediatric and young adult CHD is dominated by complex atrial tachyarrhythmias, particularly in single-ventricle physiology following Fontan palliation, Mustard/Senning atrial switch procedures, or complex atrial septal defect repairs. IART and atypical atrial flutter represent leading causes of hemodynamic collapse, thromboembolism, and sudden death in this population.

Recent advances in adult AI-ECG literature demonstrate that deep learning can detect subtle, sub-visual atrial remodeling and predict paroxysmal atrial fibrillation/flutter directly from sinus rhythm ECGs (6, 26). Translating these capabilities to CHD is paramount, as AI models trained on vectorcardiographic features can identify localized atrial conduction delay, low-voltage scar zones, and fragmented *P*-wave signatures characteristic of intra-atrial re-entry circuits in Fontan patients prior to clinical arrhythmia onset. Because Fontan patients tolerate tachyarrhythmias poorly due to loss of passive ventricular filling, AI-driven surveillance during sinus rhythm could trigger early ambulatory Holter monitoring, electrophysiological evaluation, or prophylactic antiarrhythmic therapy before overt hemodynamic decompensation occurs (6).

### AI-guided catheter ablation and electroanatomical mapping

Invasive electrophysiology is increasingly augmented by AI-driven procedural support. Advanced electroanatomical mapping systems incorporate machine learning algorithms to automatically annotate local activation times, analyze complex fractionated atrial electrograms, and differentiate true conduction scar from far-field signals in surgically altered heart anatomy. Furthermore, AI models combining pre-procedural late gadolinium enhancement magnetic resonance imaging with high-density invasive electrical mapping can identify critical Isthmus conduction lines for re-entrant tachycardias and predict long-term arrhythmia-free survival following catheter ablation.

## Wearable devices, remote surveillance, and workflows

### Critical appraisal of wearable technologies in children

Wearable technologies—including smartwatches (e.g., Apple Watch lead-I iECG), photoplethysmography (PPG) wristbands, and smart textile shirts—offer continuous ambulatory surveillance for pediatric arrhythmias such as supraventricular tachycardia (SVT) and premature ventricular contractions (PVCs) (27, 28). Remote monitoring of cardiac implantable electronic devices (CIEDs) via Bluetooth Low Energy (BLE) smartphone applications has further improved scheduled transmission compliance (94.6%) compared to legacy bedside wands (29, 30).

However, enthusiasm for pediatric wearables must be balanced against formidable technical and clinical challenges. Children engage in unpredictable physical activity, leading to continuous motion artifacts, baseline drift, and transient loss of skin contact, all of which generate high rates of uninterpretable tracings and false-positive arrhythmia alerts. Wearing continuous sensors for prolonged periods also encounters pediatric compliance hurdles related to sensory discomfort, skin irritation, and behavioral refusal. Furthermore, edge-computing algorithms capable of real-time beat-by-beat AI classification demand significant processing power, creating a sharp trade-off between battery longevity and diagnostic sampling frequency. Crucially, almost all consumer wearable arrhythmia algorithms are explicit in their regulatory clearances as being restricted to individuals aged twenty-two years or older, lacking formal FDA clearance or CE mark approval for pediatric diagnostic use.

### Realistic integration into pediatric clinical workflows

**Figure 1:**
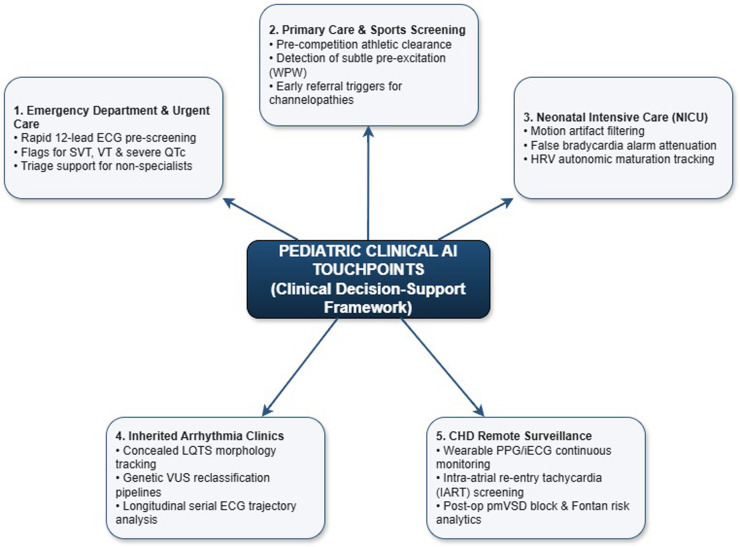
Strategic integration of artificial intelligence decision-support tools across pediatric clinical workflows. Conceptual framework illustrating the deployment of purpose-built artificial intelligence (AI) and deep learning models as clinical decision-support systems across five key pediatric healthcare environments: (1) Emergency Department & Urgent Care, providing rapid 12-lead ECG pre-screening for life-threatening arrhythmias (e.g., SVT, VT) and extreme QTc prolongation; (2) Primary Care & Sports Screening, facilitating automated detection of subtle pre-excitation (Wolff-Parkinson-White syndrome) and concealed channelopathies during routine athletic clearances; (3) Neonatal Intensive Care Unit (NICU), enabling real-time motion artifact filtering, false bradycardia alarm attenuation, and HRV autonomic maturation tracking; (4) Inherited Arrhythmia Clinics, supporting longitudinal serial ECG trajectory monitoring, concealed LQTS morphology analysis, and genomic VUS reclassification pipelines; and (5) Congenital Heart Disease (CHD) Remote Surveillance, leveraging continuous wearable telemetry for intra-atrial re-entry tachycardia (IART) screening and postoperative risk analytics. CHD, congenital heart disease; ECG, electrocardiogram; HRV, heart rate variability; IART, intra-atrial re-entry tachycardia; LQTS, long QT syndrome; NICU, neonatal intensive care unit; QTc, corrected QT interval; SVT, supraventricular tachycardia; VUS, variants of uncertain significance; VT, ventricular tachycardia.

To prevent AI from becoming an isolated technical exercise, algorithms must be conceptualized as decision-support tools integrated into specific clinical touchpoints (Figure 1). In emergency departments and urgent care centers, AI can serve as an immediate pre-screening layer on twelve-lead ECGs to flag life-threatening arrhythmias or extreme QTc prolongation prior to pediatric cardiology consultation. In primary care and sports screening environments, automated algorithms can assist non-specialists by flagging subtle pre-excitation or concealed long QT patterns during routine athletic clearances, ensuring timely referral to electrophysiologists. In neonatal intensive care units, integrated bedside alarm-filtering software can suppress mechanical motion artifacts while maintaining high vigilance for true apnea-associated bradycardia. In inherited arrhythmia clinics, AI pipelines can work alongside genetic counselors to track longitudinal T-wave changes, evaluate variants of uncertain significance, and monitor drug safety. Finally, in ambulatory congenital heart disease management, continuous wearable telemetry can provide ongoing surveillance for IART in post-Fontan patients, enabling early clinical intervention before acute hemodynamic collapse.

## Technical, ethical, and translational considerations

### Re-evaluating explainable AI (XAI): opportunities and limitations

Explainable AI (XAI) is widely advocated to solve the “black box” nature of deep neural networks. Diagnostic visualization tools, such as Gradient-weighted Class Activation Mapping (Grad-CAM) and 1D saliency heatmaps, highlight specific ECG segments (e.g., delta-wave slurring in WPW or T-wave morphology in LQTS) driving an algorithm's classification (31).

However, explainability must not be portrayed as a fully solved solution or a guarantee of clinical safety. Saliency maps are notoriously sensitive to small perturbations in input signals, where non-pathological baseline noise or minor lead motion can drastically shift heatmap concentration without changing the underlying prediction. Furthermore, saliency maps indicate statistical correlation rather than electrophysiological causality, meaning that a highlighted ECG segment does not prove the algorithm is reasoning according to established cardiac pathophysiology. Visual heatmaps can also generate a false sense of trust, leading clinicians to accept incorrect AI classifications simply because an appealing graphic is provided. Regulatory bodies increasingly demand formal verification of explainable AI reproducibility, rigorous algorithmic auditing, and clear evidence that explainability tools actually improve clinician diagnostic accuracy in prospective human-in-the-loop trials.

### Realistic short-term implementation vs. long-term horizons

To establish realistic perspectives for digital health, future research directions must distinguish short-term translational goals from long-term research horizons. Short-term implementation priorities over the next one to five years include the deployment of privacy-preserving federated learning networks across pediatric hospitals to train algorithms on diverse multicenter datasets without transferring raw patient files (32). Short-term goals also encompass prospective clinical trials evaluating real-time bedside AI algorithms designed to attenuate false alarms in pediatric intensive care units, as well as the integration of validated pediatric deep learning models into standard ECG machines to replace legacy rule-based software.

Conversely, long-term research horizons spanning five to fifteen years involve unifying twelve-lead ECGs, continuous wearable telemetry, longitudinal electronic health records, CMR imaging, and genomic data into single multi-view foundation models for holistic cardiovascular risk prediction. Long-term goals also encompass combining patient-derived human induced pluripotent stem cell cardiomyocytes with ensemble machine learning frameworks to functionally reclassify Variants of Uncertain Significance in channelopathy genes (33, 34). Finally, long-term research includes quantitative deep learning digital histopathology applied to autopsy tissue to identify sub-visual myocardial scar and micro-patterns of fatty infiltration in Sudden Arrhythmic Death Syndrome cases (35), techniques that remain highly experimental and require extensive validation prior to routine forensic application.

## Conclusions

Artificial intelligence holds transformative potential for pediatric electrophysiology, offering specialized tools for arrhythmia detection, risk stratification, and physiological monitoring. Purpose-built deep learning models have demonstrated strong diagnostic performance for specific conditions—such as concealed long QT syndrome and Wolff-Parkinson-White syndrome—in retrospective research cohorts, while predictive analytics show promise for attenuating false alarms in intensive care units and extending surveillance into the ambulatory setting via wearables.

However, enthusiasm must be balanced by the recognition that the vast majority of current pediatric AI applications remain unvalidated research constructs. AI models evaluate isolated electrocardiographic waveforms under curated conditions and cannot replace holistic clinical evaluations that synthesize patient history, family screening, imaging, laboratory data, and genetics. Realizing the translational potential of AI requires moving from proof-of-concept algorithms to safe clinical implementation. The future of pediatric electrophysiology hinges on prospective multicenter clinical validation, privacy-preserving federated learning networks to overcome data scarcity, robust explainability frameworks, and the seamless integration of multimodal AI into real-world clinical workflows as human-in-the-loop decision-support tools.
